# Offline Optimization of the Relative Timing of Movements in a Sequence Is Blocked by Retroactive Behavioral Interference

**DOI:** 10.3389/fnhum.2016.00623

**Published:** 2016-12-20

**Authors:** Jason Friedman, Maria Korman

**Affiliations:** ^1^Department of Physical Therapy, Sackler Faculty of Medicine, Tel Aviv UniversityTel Aviv, Israel; ^2^Sagol School of Neuroscience, Tel Aviv UniversityTel Aviv, Israel; ^3^Department of Occupational Therapy, Faculty of Social Welfare and Health Sciences, University of HaifaHaifa, Israel

**Keywords:** learning, interference, consolidation, finger movements, kinematics

## Abstract

Acquisition of motor skills often involves the concatenation of single movements into sequences. Along the course of learning, sequential performance becomes progressively faster and smoother, presumably by optimization of both motor planning and motor execution. Following its encoding during training, “how-to” memory undergoes consolidation, reflecting transformations in performance and its neurobiological underpinnings over time. This offline post-training memory process is characterized by two phenomena: reduced sensitivity to interference and the emergence of delayed, typically overnight, gains in performance. Here, using a training protocol that effectively induces motor sequence memory consolidation, we tested temporal and kinematic parameters of performance within (online) and between (offline) sessions, and their sensitivity to retroactive interference. One group learned a given finger-to-thumb opposition sequence (FOS), and showed robust delayed (consolidation) gains in the number of correct sequences performed at 24 h. A second group learned an additional (interference) FOS shortly after the first and did not show delayed gains. Reduction of touch times and inter-movement intervals significantly contributed to the overall offline improvement of performance overnight. However, only the offline inter-movement interval shortening was selectively blocked by the interference experience. Velocity and amplitude, comprising movement time, also significantly changed across the consolidation period but were interference –insensitive. Moreover, they paradoxically canceled out each other. Current results suggest that shifts in the representation of the trained sequence are subserved by multiple processes: from distinct changes in kinematic characteristics of individual finger movements to high-level, temporal reorganization of the movements as a unit. Each of these processes has a distinct time course and a specific susceptibility to retroactive interference. This multiple-component view may bridge the gap in understanding the link between the behavioral changes, which define online and offline learning, and the biological mechanisms that support those changes.

## Introduction

### Motor Sequence Learning

Motor sequence learning refers to the ability to create a link between temporal events, consisting of actions or movements (Adini et al., 2015) and involves transforming a number of discrete movements that are serially executed into a merged representation encompassing multiple anticipated movements, a process known as chunking (Karni, 1996; Gobet et al., 2001; Verwey, 2003; Verwey and Eikelboom, 2003; Sosnik et al., 2004). Skillful performance of motor sequences is involved in almost every aspect of our everyday activities: from typing to language to social behaviors. A basic assumption suggests the existence of an underlying “motor plan” at different abstraction levels (Chafee and Ashe, 2007), a plan that articulates simple movements into novel sequences that are executed faster and smoother with practice.

Sequences can be learned in different ways: explicitly, e.g., in finger-opposition or finger-tapping paradigms (Korman et al., 2003; Doyon et al., 2009b; Friedman and Korman, 2012) or implicitly, e.g., in serial reaction time paradigms (Destrebecqz and Peigneux, 2005; Janacsek and Nemeth, 2012), depending on the intention or awareness during acquisition (Nissen and Bullemer, 1987; Hikosaka et al., 2002; Robertson, 2007). Both types of sequence learning depend on the amount of practice (Karni, 1996; Schmidt and Lee, 1999; Page and Norris, 2009). Practice at its simplest is just performing the same movements repeatedly. Although this is a necessary prerequisite of improving performance during the training session, it is neither optimal nor sufficient for the establishment of the skill or the retention of the learning over time (Korman et al., 2003). Consistency in experiencing the specific sequence was shown to be critical for the optimal formation of long term memory. When consistency is violated, for example, by experiencing a similar but different movement sequence shortly after practicing the first sequence (retroactive interference), the long-term learning of the sequence is hampered (Korman et al., 2003, 2007). Additional factors such as self-guided performance (Friedman and Korman, 2012), structural specificity (Rozanov et al., 2010) and affordance of post-training sleep (Walker et al., 2003; Korman et al., 2007) were shown to affect the resultant long term memory for a given motor sequence.

### Time-Course of Sequence Learning

Numerous studies have examined the time course of learning sequences of finger movements on a basic behavioral level (number of sequences performed, error rate) as well as neural correlates using neuroimaging (Karni et al., 1995; Hikosaka et al., 2002; Keele et al., 2003; Korman et al., 2003; Luft and Buitrago, 2005; Janacsek and Nemeth, 2012). A few hours after practice, skills are processed, changed and strengthened in memory (Karni and Sagi, 1993; Karni et al., 1995), a phenomenon commonly described as procedural memory “consolidation” (Karni and Sagi, 1993; Walker, 2005). The consolidation phase reflects processes with qualitative (Korman et al., 2003), structural (Yang et al., 2014) changes in the neuronal circuitry engaged in task representation.

Behaviorally, consolidation refers to the maintenance or enhancement of performance over an interval including no further practice - off-line, between practice sessions improvement (delayed, “offline” gains); and the increase in stability of a novel memory trace, making it no longer susceptible to interference (Brashers-Krug et al., 1996; Walker, 2005; Dudai, 2012; Robertson, 2012; Albouy et al., 2015). Considerable evidence suggests that sleep favors the development of the first behavioral determinant and consequently facilitates motor sequence memory retention as compared to wakefulness (Walker et al., 2002; Robertson et al., 2004; Nettersheim et al., 2015). Motor skill consolidation benefits from both night-time and day-time sleep (Korman et al., 2003, 2007; Walker and Stickgold, 2010; Tononi and Cirelli, 2014; Yang et al., 2014). Both aspects of consolidation processes, the delayed gains in performance and robustness to retroactive interference, are non-mutually exclusive phenomena and can be measured in laboratory settings in the context of motor sequence skill learning (e.g., Korman et al., 2003, 2007).

It is often desirable that a newly learned skill can also be applied within a different task or context. Susceptibility to transfer is thus an important aspect of motor learning (Müssgens and Ullén, 2015) and can be divided into sequence-specific, attributed to knowledge of the sequential order of the task elements (Park and Shea, 2005; Verwey and Clegg, 2005) and sequence non-specific components. How the processes of acquisition and transfer of a learned skill are interrelated is an important issue that needs to be investigated to enable development of training protocols whereby training can predict generalization of the acquired knowledge (Speelman and Kirsner, 2001). Training schedule, explicit vs. implicit nature of knowledge, broad vs. narrow transfer demands may critically affect the ability to generalize the sequential skill (Müssgens and Ullén, 2015). In particular, transfer between hands, in terms of number of correct sequences performed, is likely to be positive for sequence-specific as opposed to non-specific conditions (Grafton et al., 2002; Korman et al., 2003).

Motor sequence learning involves a complex interaction between at least two cerebral systems, i.e., cortico-striatal and cortico-hippocampal networks (Albouy et al., 2013). According to this model, these different systems would support separate but interacting consolidation processes. The hippocampal system favors sleep-dependent motor memory consolidation processes, whereas the striatal system appears to support more of a time-dependent processing of the memory trace (Albouy et al., 2015), that may nevertheless be modulated by sleep via interactions with the hippocampal system (Albouy et al., 2013). Altogether, successful retention of motor sequence memories is presumably supported by a cooperative interplay between the striato-motor and hippocampo-cortical networks (Albouy et al., 2008, 2013).

### Retroactive Interference

Retroactive interference relates to the disruptive effect of experience subsequent to the learning session on the ability to express the expected consolidation performance gains. The time-window of potential retroactive interference is short and may be suspended following initial stabilization (robustness to interference), which has been shown to take effect within a few hours proceeding initial training (Brashers-Krug et al., 1996; Korman et al., 2003; Balas et al., 2007a,b) and possibly earlier if a short nap (Korman et al., 2007) follows the initial practice. Recently, we have shown using a paradigm of retroactive interference that cortico-striatal areas play a critical role in the sleep-facilitated reduction in motor memory vulnerability (Albouy et al., 2016). It was suggested that the magnitude of the effect of behavioral interference is critically dependent on an overlap between the representations of the two tasks, and that such overlap is more likely when the two tasks are novel, competing for general resources for their execution (Tong et al., 2002; Miall et al., 2003; Krakauer et al., 2005). Surprisingly, the kinematic and temporal correlates of retroactive interference to motor sequence memory consolidation and the ability to generalize the acquired knowledge across effectors have never been systematically characterized. This knowledge gap is striking, as understanding how the brain deals with the plethora of competition between motor tasks in everyday life, which often share common attributes, is of the utmost importance.

### Kinematic and Temporal Correlates of Sequence Learning

There have been few studies looking at how finger-sequence learning is achieved, or affects movement properties, on a kinematic level (Orban et al., 2011; Friedman and Korman, 2012). In the literature it is recognized that two general aspects of performance are being optimized through the course of learning: (1) the overall temporal structure of the acquired sequence, known as coarticulation (Jerde et al., 2003; Sakai et al., 2004), presumably reflecting a higher-order representation of the sequence - a “motor plan” (Hikosaka et al., 2002), and (2) the kinematic properties of the movements (Orban et al., 2011; Friedman and Korman, 2012), presumably contributing to an enrichment of the amount of sensory feedback available during task performance, thus supporting bottom-up learning processes (Drewing et al., 2002). Both contribute to the emergence of a novel, sequence specific representation of the trained task. Motor learning is usually quantified by an improvement in the speed accuracy trade-off (Shmuelof et al., 2012). Distinct brain areas were demonstrated to be involved in the associated kinematic and temporal changes, including cerebellar-cortical and striatal-cortical motor networks (Ungerleider et al., 2002; Lehéricy et al., 2005; Doyon et al., 2009a; Orban et al., 2011; Albouy et al., 2015).

Learning in sequential finger tasks is usually measured using general variables: movement time for quantifying movement difficulty, and quality of performance for quantifying accuracy. However, ascertaining whether improved movement time has been acquired can be problematic if factors accounting for movement time, e.g., amplitude of movements and velocity of movements, change in opposite directions during the course of learning, masking each other. In other words, it is plausible that some learning-related changes in the motor output are missed when only gross performance measures of movement times or whole sequence time are assessed in the analysis. One way to overcome this problem is to record the movements in more detail, i.e., the kinematics. In a previous study (Friedman and Korman, 2012), using a keyboard sequence task, we showed that long-term improvements in the number of correct sequences performed were mainly due to reducing inter-movement intervals between finger movements. There was also an overall reduction in movement time, but it was small because while subjects increased the speed of their movements, they concurrently increased their movement amplitude. Presumably, touching the keys with a greater force enables enhancement of the tactile feedback received during practice (Shadmehr et al., 2010). This sensory reafference may help improve timing accuracy (Drewing et al., 2002).

### The Current Study

The present experiments investigated how specific temporal and kinematic parameters of sequence performance consolidate over a 24 h time-window following training. In particular, we assessed which aspects of the movements comprising a trained sequence are sensitive to retroactive interference and which consolidate independently of post-training intervention. Our apparatus and analytical approach allowed disentangling multi-finger sequence completion time into individual finger’s movement time, touch time, inter-movement interval, velocity and amplitude, to achieve a better resolution of analysis compared to more traditional methods.

We tested the possibility that higher-order learning-related processes will be selectively disrupted by additional training on a sequence with a different ordinal structure, while other major contributors to improvements in performance, e.g., kinematic features of individual movements will be insensitive to such experience. We hypothesized that behavioral retroactive interference will have a significant impact on the time-course and magnitude of changes in distinct temporal and kinematic features of the originally trained tasks and transfer tasks. We predicted that this will be in addition to previously reported changes in speed and accuracy of performance, reflecting qualitative rather than just quantitative changes in task representation when a competing experience is afforded shortly after the training. Thus, using a well-established sequence learning paradigm that effectively induces procedural memory consolidation (the finger opposition sequence task), we investigated the effects of interference afforded within 2 h after the training session on the acquisition and memory consolidation of a motor skill over a 24 h post-training time window. To probe the nature of the internal representations presumably subserving the gains in performance triggered by the training session, the ability of participants to generalize their experience under different task conditions was assessed on the following day. The protocol was as performed in Korman et al. (2003), but with the addition of recordings of kinematics (fingertip movements). We observed the within- and between- session changes in performance of a trained sequence of finger movements over two consecutive days in two conditions: with and without additional sequence training (retroactive interference). We examined two main questions: which changes in motor parameters will be affected by the interference protocol, and how do the interference-dependent and interference-resistant parameters generalize across sequences and hands.

## Materials and Methods

### Participants

Thirty-four right-handed subjects (24 females, average age 24 ± 2, range 20–29) from the Tel Aviv University student population took part in the experiment. Right-handedness was confirmed using the Edinburgh Handedness Inventory (Oldfield, 1971). Ethics approval was received from the Tel Aviv University Human Ethics committee, and the participants signed an informed consent form before beginning the experiments. Subjects were paid 70 shekels (approximately $20) for their participation.

### Experiment Protocol

**FIGURE 1:**
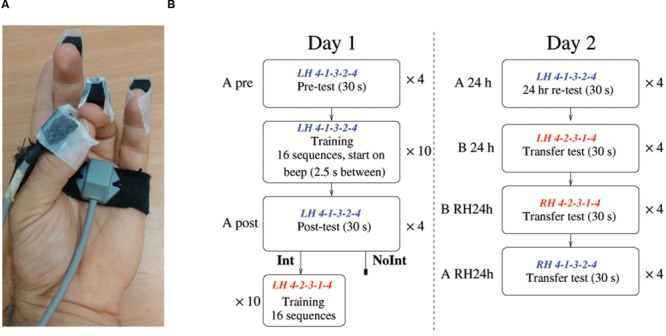
**Experimental setup and protocol. (A)** The task involved touching the finger (1 = index, 2 = middle, 3 = ring, 4 = little) with the thumb in the specified order. Polhemus liberty markers were taped to the fingertips and to the palm of the hand to record the relative movements of the fingers. **(B)** The experimental protocol over the 2 days of the experiment. The initially trained sequence A (4-1-3-2-4, blue) and the sequence B (4-2-3-1-4, red), used for interference training, were composed of the same movements, but in the opposite order. Note that the two groups only differed in the interference experience – while the interference group (Int) received an additional training session on the B sequence (shown in red) 30–90 min after the end of the training in A sequence, the no interference group (NoInt) did not.

The participants were required to perform a finger opposition task (Korman et al., 2003), where the thumb and another finger of the left, non-dominant hand are required to touch in a given sequence (4-1-3-2-4), (1) corresponded to the index finger touching the thumb, (2) to the middle finger touching the thumb, etc. (see **Figure 1A**). The experimenter demonstrated touching the thumb and finger, but did not demonstrate the sequence. The participants were instructed not to look at their fingers while performing the task. In the first session, the participants first performed four test trials (A pre). Each trial was 30 s long, and the participants were instructed to perform accurately as many sequences (4-1-3-2-4) as possible during this time. Beeps indicated the start and end of the trial, and 1 min rest was provided between trials. Before each trial, the sequence was shown on the screen as text (e.g., 4-1-3-2-4), during the performance the screen was blank.

Following this, the participants performed 10 training trials. In each trial, there was a beep every 2.5 s. The participants were instructed after hearing each beep to perform the sequence (A: 4-1-3-2-4) once, with a focus on accuracy. There were 16 repetitions of the sequence in each training trial, with 30 s rest between trials. The sequence was shown on the screen at the beginning of each trial during the training, but not while the movements were being made. Following the training, subjects performed again four test trials (A post), with identical instructions to the initial tests.

Half the subjects [Interference group (Int): *N* = 17] returned to the lab between 30 and 90 min later to perform the interference training, on a different sequence, comprised of the same movements as the first sequence, but in a different order (B: 4-2-3-1-4). No tests were performed during this session. The other half of the subjects did not perform any additional trials on that day [No interference group (NoInt): *N* = 17].

Twenty-four hours after the initial training session, subjects were tested in the performance of the trained sequence A and of three transfer conditions, to assess the transfer capability, following the same instructions as used on the 1st day test. First, the participants were afforded a test on the original sequence 4-1-3-2-4 (A 24 h), followed by the second sequence 4-2-3-1-4 (B 24 h), using their left hand. Following this, they also performed both sequences with the right hand: first the second sequence (B 24 h RH) then the first sequence (A 24 h RH). The protocol is summarized in **Figure 1B**.

### Measurement and Data Pre-processing

The finger movements of the participants were recorded using a Polhemus Liberty magnetic motion capture system, sampling at 240 Hz. The locations and orientations of six sensors were recorded, one taped on each fingertip and one on the palm of the left hand. For the last two blocks, where the right hand was tested, the sensors were moved to equivalent locations on the right hand. The sensors were taped on the fingertips such that no tape was on the finger pads (to preserve full touch sensation at the fingertips). The experiments were run using the “Repeated Measures” software (Friedman, 2014), Matlab (Mathworks, Inc.) software that runs on top of the Psychtoolbox (Brainard, 1997).

**FIGURE 2:**
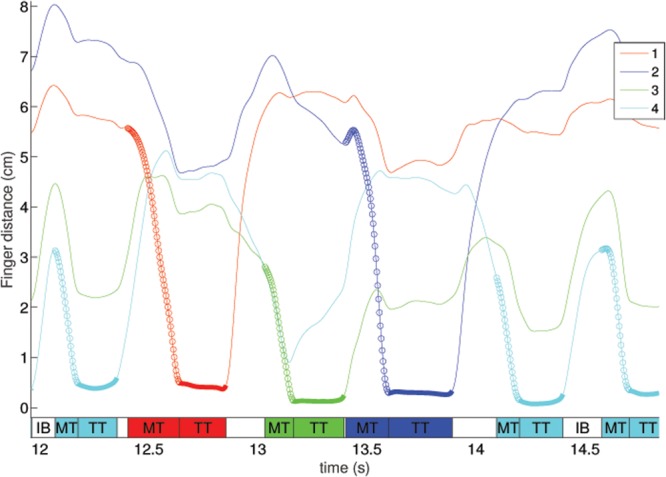
**An example of the decomposition of the movements into the various components, from a representative subject.** The graph shows the distance between the thumb and the specified finger. The sections marked with circles are defined as the movement time (MT), and the sections marked with a thicker line are the touch times (TT). The colored rectangles at the bottom of the figure refer to the phases of the sequence acting along sequence execution. The time after the conclusion of the touch and the start of the next movement is defined as the inter-movement intervals, either within a sequence (IW – the unlabeled white rectangles at the bottom of the figure) or between a sequence (IB).

Data were analyzed offline using custom Matlab software. Finger touches (of the thumb and other finger) were identified automatically based on the minima of distances between the thumb and other fingers. The timing of these touches was manually corrected so that the number of sequences and errors performed matched the number recorded by the experimenter (verified from video-recordings). Each sequence was then decomposed to determine the relative contribution of the different parts, following the technique used in Friedman and Korman (2012). An example of the decomposition is shown in **Figure 2**. The calculations were performed on the distance between the thumb and relevant finger. Specifically, the time of each sequence was broken down into the time of the finger movement (from the last trough in the derivative of the finger distance; i.e., when the finger and thumb start moving closer together to the moment they touch), to the inter-movement intervals, between movements within a sequence, and between sequences (i.e., between the end of one sequence and the start of the next sequence). The touch times (when the thumb was touching the other finger) were defined as the time adjacent to the touch where the magnitude of the derivative of the finger-thumb distance is below 5% of the maximum magnitude of the derivative of the finger-thumb distance. Normalized data (relative improvement) was calculated relative to baseline performance in the first two trials of the pre-test, described in more detail in Friedman and Korman (2012). We performed this normalization on the time taken to correctly complete a sequence, and on the components described above (movement times, touch times, inter-movement intervals). Specifically, we subtracted the duration of the appropriate component from the baseline value (for one sequence), then divided it by the duration of the sequence, and multiplied by 100 to get a percentage. For right-hand and sequence B test trials, the relative improvement was calculated using the same baseline as all the other trials (i.e., sequence A, left hand). This is based on previous studies which have shown that both sequences are of approximately equal difficulty and there is no difference in baseline performance between the two sequences and the two hands (Korman et al., 2003; Dorfberger et al., 2007, 2009).

We also computed the peak velocity and the amplitude of the movements. These calculations are performed relative to the coordinate system of the hand. The peak velocity was defined as the maximum velocity of the finger (not the thumb) during the finger movement preceding the touch (as described above in the timing parameters). The amplitude of the movement was defined as the distance from the position of the finger at the start of the finger movement, to the position of the finger at the touch. Both peak velocities and amplitudes were averaged across fingers and repetitions (within a 30 s test).

### Statistical Analysis

We analyzed separately three stages involved in learning, consolidation and transfer. The first is the fast learning that takes place during the training session. We performed a paired sample *t*-test with factor session (A pre vs. A post). We performed this analysis on the number of correct sequences, as well as on the decomposition into the four components described above, and the peak velocity and amplitude.

We then measured the changes in overnight consolidation with a mixed design ANOVA between the two groups, with factor session (A post vs. A 24 h), examining the number of correct sequences/normalized improvement, as well as the decomposition into the four components described above, and peak velocity and amplitude. We performed the analyses for both absolute values as well as the normalized data. Previous studies looking at sequence learning have sometimes used absolute values (e.g., Korman et al., 2003), and sometimes normalized values (e.g., Ossmy and Mukamel, 2016). This makes it difficult to compare between studies. To allow better comparison with other studies, and to demonstrate that the results are not due to using normalization (or not), we included both normalized and non-normalized values for the primary findings of the study.

To test the effect of the interference experience on the different types of transfer, we performed a mixed design ANOVA on the normalized data between the two groups, with within-subject factors of sequence (A and B), and hand (LH and RH), i.e., performed on the four tests performed on the 2nd day. Due to differences in baseline performance between the groups, we will only perform this analysis for normalized data. Similarly, this analysis was performed both on the overall normalized improvement, as well as on the normalized improvement of the four components.

Within-session learning was quantified by looking at the novelty effect, defined as the slopes of linear regression lines that were fitted individually to the non-normalized data for each of the four test blocks, with a steeper slope indicating greater novelty. The linear regression lines were fitted to each subject, and then individual slope coefficients were averaged (by group).

For the ANOVAs, we used a significance level of *p* < 0.05. Paired *t*-tests were used to help explain the observed interactions. All values are presented as means ± standard error.

## Results

We present the results for the three stages of learning, consolidation and transfer. For each stage, we present the results on the overall number of sequences/normalized improvement, followed by a decomposition into four components: movement times, touch times and both within- and between-sequence inter-movement intervals.

### Training

#### Training – Number of Sequences

**FIGURE 3:**
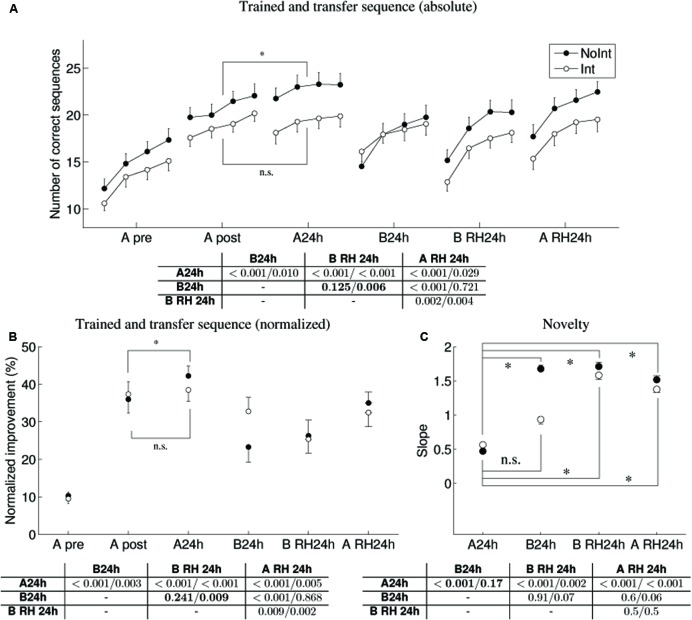
**Performance of trained and transfer sequences across the 24 h interval (pre-test, post-test, 24 h)for the NoInt and Int groups (A)** Absolute performance (number of correct sequences), note that only the NoInt group shows significant overnight consolidation gains. **(B)** Normalized performance (relative to the first two tests of the pre-test, averaged across four performance trials) replicate the statistical conclusions as in **(A)**. **(C)** Novelty effect (block-by-block changes in performance) for the transfer conditions. Data points are the mean slopes of linear regression lines, fitted to the four blocks of each test condition. Higher values indicate higher novelty. Tables of non-corrected pairwise comparisons between performances of NoInt/Int groups at 24 h post-training in three test conditions are shown for the absolute, normalized and novelty data, respectively. Note, that following interference training, at 24 h tests, the difference in the representation of sequence A and sequence B are evident both in the number of correct sequences and the novelty measures.

The training session was effective: the participants increased in the number of sequences they performed (see **Figure 3A**), from an average of 14.2 (±0.7) to 19.8 (±0.7) sequences (averaged over the 4 pre-tests compared to the 4 post-tests). This increase in the number of sequences is supported by a significant paired sampled *t*-test [*t*(33) = -12.6, *p* < 0.001]. We note that the same effect is observed for both groups, if considered individually [NoInt: increase from 15.1 ± 1.0 to 20.8 ± 1.1, *t*(16) = -8.83, *p* < 0.001; Int: increase from 13.3 ± 1.0 to 18.8 ± 0.9, *t*(16) = -8.69, *p* < 0.001], showing that there is no a priori differences in learning between the groups. Note that the absolute errors were minimal and showed a small increase with training [average of 1.6 ± 0.3 and 2.5 ± 0.4, *t*(33) = -2.09, *p* = 0.045, pre-test and post-test, respectively], likely due to the increase in number of sequences performed. The errors did not show any significant changes with consolidation, so will not be analyzed further.

#### Training – Components

**FIGURE 4:**
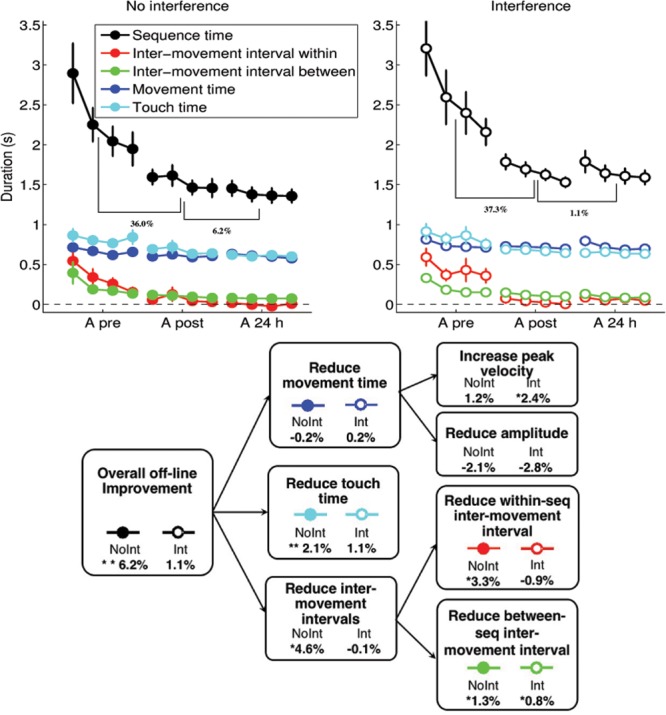
**Decomposition of the sequence time (the time taken to perform a sequence) into its components: inter-movement intervals, movement times and touch times.** Within session and between session relative improvement are shown as percentage. The diagram shows the decomposition for both groups into three major factors contributed to overall offline reduction (improvement) in sequence time: (i.e., movement time, touch time and inter-movement interval reductions), and their further decompositions. The values show the difference between the 24 h test and post-test, with positive values indicating greater improvement at 24 h post test. ^∗^indicates 0.05 < *p* < 0.1, ^∗∗^indicates *p* < 0.05.

**FIGURE 5:**
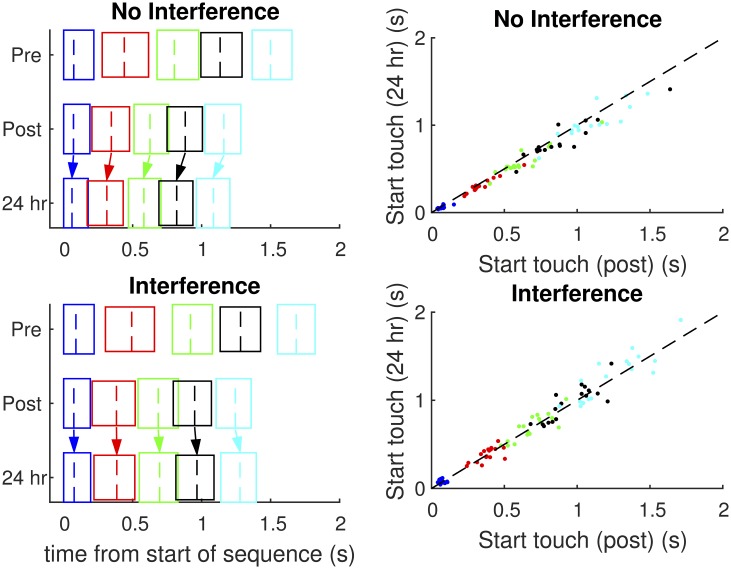
**Timing of the sequences.** The left two panels show the mean times of the individual finger movements comprising the sequence for the two groups. The left solid vertical line of each box indicates the start of the movement, the dashed line indicates the start of the touch, and the right vertical line indicates the end of touch. Overlapping boxes indicate coarticulation (i.e., the later movement starts before the previous finger has finished touching). The scatterplots show the relationship between the mean touch times at post-test and 24-h retest, per individual finger. The dashed line represents the same time for both the tests. Note that for the NoInt group, nearly all the dots are below the dashed line (i.e., faster at 24 h retest), while this is not the case for the Int group.

**FIGURE 6:**
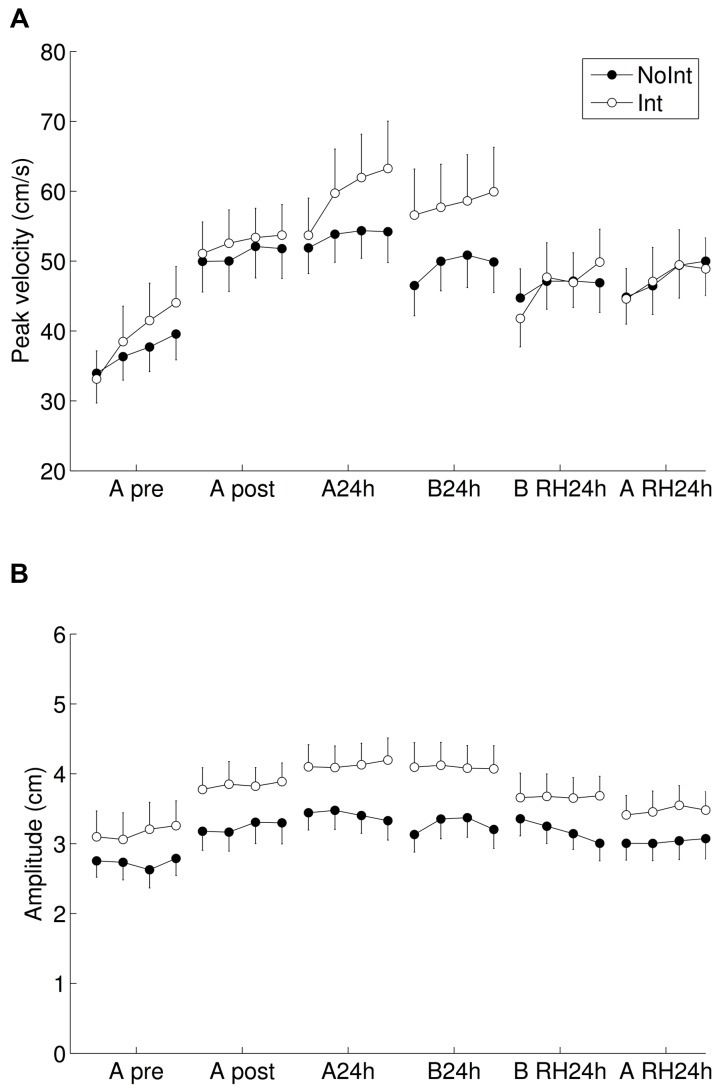
**Peak velocity and amplitude of the movements.** The values shown are the **(A)** peak velocity and **(B)** amplitude of the finger (not the thumb) during the closing movement directly preceding the touch, averaged across the five movements in the sequences. We note that while relatively large increases in peak velocity are observed, the concurrent increase in amplitude leads to very small changes in the movement time.

As can be observed in **Figure 4** (upper panel), the bulk of the improvement comes from the reduction in the inter-movement intervals within a sequence and between sequences. By the end of the post-test (within-session), the within-sequence inter-movement intervals reduced to 51 ± 31 ms, which was not significantly different from 0 [*t*-test: *t*(33) = 1.61, *p* = 0.12]. The average times of the performance of the different finger movements are shown in left panels in **Figure 5**. Initially, subjects make individuated movements, finishing one movement before starting the next (Pre-test, both groups). With practice, the movements start to overlap – subjects begin making the next movement while the fingers are still touching in the previous movement. This can be seen as overlapping rectangles at Post-test in both groups in **Figure 5**. A smaller but significant reduction is observed in the touch times, from 865 ± 60 ms to 672 ± 32 ms [*t*(33) = 4.84, *p* < 0.001] and the movement times, from 705 ± 25 ms to 661 ± 22 ms; [*t*(33) = 4.84, *p* = 0.0496]. While only a small overall reduction in movement times was observed, the magnitude of changes in peak velocity and amplitude, contributing to changes in movement times was much larger, as is shown in **Figure 6**. Specifically, peak velocity (averaged across fingers) increased from 38.1 ± 2.8 cm/s to 51.8 ± 3.0 cm/s; [*t*(33) = -6.75, *p* < 0.001], and amplitude increased from 2.94 ± 0.22 cm to 3.54 ± 0.20 cm; [*t*(33) = -3.82, *p* = 0.001]. As these effects work in opposite directions (an increase in amplitude will lead to slower movements), they practically canceled each other; this explains the much smaller relative reduction observed when looking at movement times.

### Overnight Consolidation

#### Overnight Consolidation – Number of Sequences and Normalized Improvement

We next compared the overnight consolidation to test whether there is an improvement in performance between the post-tests and the tests at 24 h. Here we considered the two groups separately, as the Int group experience an additional training session on the B sequence. While there was a main effect of session [*F*(1,32) = 24.4, *p* = 0.002], we will focus on the significant interaction of session and group [*F*(1,32) = 5.2, *p* = 0.029]. This interaction is due to the finding that only for the NoInt group were additional robust gains in the number of correct sequences observed [*t*(16) = -3.72, *p* = 0.002], see **Figure 3A**, with an improvement from 20.8 (±1.08) to 22.8 (±1.2) sequences. The Int group did not show a significant improvement. A similar time-course can be observed in the normalized data (**Figure 3B**), although the interaction is close to significant [*F*(1,32) = 3.6, *p* = 0.066]. Similarly, with the normalized data we find significant improvement only in the NoInt group [*t*(16) = -2.99, *p* = 0.009], with an improvement from 36.0 (±15.0)% to 42.2 (±11.0)%, relative to baseline. Interference training experienced following the initial training prevented the expression of delayed gains in the normalized improvement at 24 h post-training for the Int group [*t*(16) = -0.74, *p* = 0.47].

#### Overnight Consolidation – Components

Differences between the groups were observed in the overnight (offline) learning, depending on the affordance of the interference training on sequence B. The lower panel in **Figure 4** shows the difference in normalized improvement between the post-test, and the 24 h retest. As described above, only the NoInt group shows significant offline gains in mean sequence duration (6.2%) (**Figure 4**, lower panel). This improvement was achieved by a combination of a reduction in touch times, and inter-movement intervals. We performed a mixed design ANOVA on the four quantities (within-sequence inter-movement intervals, between-sequence inter-movement intervals, touch times and movement times) to determine the differences between the groups.

With overnight offline consolidation, a further optimization of within-sequence timing is observed, but only for the NoInt group. Specifically, in the NoInt group, the inter-movement intervals within a sequence, including the finger pairs 4-1,1-3, 3-2, 2-4 (shown in red in **Figure 4**) actually become negative for some of the subjects, which is an indication of coarticulation (the next movement begins before the fingers stop touching). While there was no main effect, there was a significant interaction of group and test [*F*(1,32) = 5.97, *p* = 0.02], due to the reduction seen in the NoInt group [post-test: 65 ± 45 ms; 24 h: 0 ± 36 ms. *t*-test: *t*(16) = 2.26, *p* = 0.04] but not in the Int group [post-test: 36 ± 45 ms; 24 h: 65 ± 36 ms. *t*-test: *t*(16) = -1.13, *p* = 0.28]. In the normalized data, the interaction was close to significant [*F*(1,31) = 3.85, *p* = 0.059], and similar differences were seen between the groups: NoInt group [post-test: 12.1 ± 2.4%; 24 h: 15.4 ± 2.0%. *t*-test: *t*(16) = -1.79, *p* = 0.093], Int group [post-test: 16.4 ± 2.5%, 24 h: 15.5 ± 2.1%, *t*-test: *t*(15) = 0.89, *p* = 0.386].

This reduction of inter-movement intervals within a sequence accounts for approximately half the overall offline improvement in sequence duration. This difference is further highlighted in the right panels of **Figure 5**, which compare the start of the touch time for five elements of the sequence, compared between the post-test and 24 h retest. Values below the dotted line indicate an improvement (faster) performance at 24 h retest. While for the NoInt group (upper right graph), most subjects / fingers were indeed faster, this is not the case for the Int group (lower right graph).

Whereas the reduction of inter-movement intervals within a sequence was sensitive to interference, other measures of performance were insensitive to the interference experience, and showed similar changes across the consolidation window. While overnight consolidation was observed for between sequence inter-movement intervals (i.e., the finger pair 4-4), significant differences were not found between the groups, rather only a main effect of test was observed [*F*(1,32) = 10.484, *p* = 0.003], due to a reduction in between-sequence inter-movement interval from 110 ± 7 ms to 88 ± 6 ms. The normalized data showed a similar effect [increase from 5.1 ± 0.8% to 6.1 ± 0.8%, *F*(1,31) = 6.31, *p* = 0.017]. There was no main effect of group or interaction.

The touch times similarly showed a main effect only of test [*F*(1,32) = 6.86, *p* = 0.013], with an overnight reduction in touch times from 672 ± 32 ms to 627 ± 31 ms. The normalized data also showed a significant effect on touch times [increase from 1.5 ± 0.2% to 1.8 ± 0.2%, *F*(1,31) = 7.42, *p* = 0.010]. No main effect was observed for group, nor the interaction [*F*(1,32) = 1.03, *p* = 0.32].

In terms of the kinematics of the movements, there was no main effect on movement time due to consolidation [*F*(1,32) = 0.12, *p* = 0.73]. There was also no interaction, although a main effect is observed for the group [*F*(1,32) = 7.53, *p* = 0.01], likely due to baseline differences in movement times between the groups – this main effect is not observed in the normalized data [*F*(1,31) = 0.39, *p* = 0.539].

Although we did not observe overnight consolidation changes in the measure of movement time, we tested whether the movements themselves changed, by looking at the peak velocity and amplitude, see **Figure 6**. With overnight consolidation, we see that the peak velocities increased from 51.9 ± 3.0 cm/s to 56.6 ± 3.6 cm/s [*F*(1,32) = 6.06, *p* = 0.019], but again no main effect was observed for group, nor the interaction [*F*(1,32) = 1.25, *p* = 0.27]. The amplitudes also showed robust consolidation changes, with an increase in amplitude from 3.54 ± 0.20 cm to 3.77 ± 0.20 cm [*F*(1,32) = 4.45, *p* = 0.04], with no main effect of group or interaction [*F*(1,32) = 0.27, *p* = 0.60]. We note that an increase in amplitude increases rather than decreases the movement time, as the path of the movement becomes longer. These two opposing effects, the increase in peak velocities and the increase in movement amplitudes, cause there to be no significant change in movement time.

### Transfer

#### Transfer – Normalized Improvement

We now consider the transfer conditions measured on the 2nd day. Specifically, we are interested in whether there is transfer to a second sequence (B), to the other hand (RH), and whether this differs between groups. To test this, we performed a mixed design ANOVA on the normalized data, with the ANOVA results summarized in **Table 1**. The tables at the bottom of **Figure 3** show the pairwise comparison for both groups.

**Table 1:** Results of the mixed design ANOVA examining the transfer conditions.

Factor	*F*(1,32)=	*p*
Sequence (A/B)	54.2	<0.001*
Hand (L/R)	14.4	0.001*
Group (Int/NoInt)	0.02	0.889
Group × Sequence	7.24	0.011*
Group × Hand	4.05	0.053
Hand × Sequence	4.90	0.034*
Group × Hand × Sequence	8.76	0.006*

*The dependent variable was the normalized improvement. Within-subject factors were sequence (A or B) and hand (left or right), with a between subject factor of group (Int or NoInt). Significant results (p < 0.05) are marked with an asterisk (^∗^).*

The ANOVA showed that overall, there was incomplete transfer to the second sequence (A: 37.0 ± 2.1% vs. B: 27.0 ± 2.6%), indicating a qualitative change in the representation of the task within 24 h post-training. There was also incomplete transfer from the left hand to the right hand (LH: 34.2 ± 2.3% vs. RH: 29.8 ± 2.4%). However, the hand by sequence interaction showed that for the untrained hand (RH), there was less difference between sequences (A: 28.1 ± 2.8% vs. B: 25.8 ± 2.8%) than for the trained hand (A: 40.3 ± 2.0% vs. B: 33.7 ± 2.4%).

A significant interaction of group and sequence was observed. This is because the NoInt group showed less transfer to the B sequence (A: 38.5 ± 3.0% vs. B: 24.8 ± 3.7%) compared to the Int group (A: 35.7 ± 3.2% vs. B: 28.9 ± 3.5%).

A three-way interaction of group, hand and sequence was observed. For the NoInt group, we found a significant difference for the A sequence between the trained and untrained hand [LH: 42.1 ± 2.9% vs. RH: 34.9 ± 3.4%, *t*(16) = 4.38, *p* < 0.001], but not for B [LH: 23.3 ± 3.9%, RH: 26.3 ± 4.0%, *t*(16) = -1.21, *p* = 0.24]. In contrast, for the Int group, we see a difference for both the A sequence [LH: 38.5 ± 2.9% vs. RH: 32.5 ± 3.4, *t*(16) = 3.29, *p* = 0.005] and the B sequence [LH: 32.9 ± 3.9% vs. RH: 25.4 ± 4.0%, *t*(16) = 2.96, *p* = 0.009].

#### Transfer – Normalized Components

A mixed design ANOVA was performed on the four components that contribute to the overall improvement in performance. The results of the ANOVA can be found in **Table 2**. Here, we will focus only on the significant effects. For sequence, we observe that transfer to the B sequence was incomplete in all four measures, i.e., there was less improvement in all measures (within-sequence inter-movement intervals: 13.1 ± 1.5% vs. 7.7 ± 1.5%; between-sequence inter-movement intervals: 6.2 ± 0.8% vs. 5.3 ± 0.8%; movement times: 3.4 ± 1.1% vs. 2.2 ± 1.0%; touch times: 1.7 ± 0.2% vs. 1.5 ± 0.2%). While there was an overall significant effect of the hand used on the number of correct sequences (see above), for the components, we only observe a significant effect for within-sequence inter-movement intervals (LH: 12.0 ± 1.4% vs. RH: 8.8 ± 1.6%). Similarly, the only significant interaction of group and sequence was for the within-sequence inter-movement intervals: the difference between A and B was smaller for the Int group (A: 12.9 ± 2.1%, B: 8.8 ± 2.1%) than for the NoInt group (A:13.3 ± 2.0%, B: 6.6 ± 2.1%). The within-sequence inter-movement intervals also showed a significant interaction for the interaction of hand and sequence – less difference was observed between the sequences for the right hand (A: 10.7 ± 1.8%, B: 6.9 ± 1.6%) than for the left hand (A: 15.5 ± 1.4%, B: 8.5 ± 1.5%). Touch times also showed a significant interaction for hand and sequence, with the touch times only different for the left hand [A: 1.8 ± 0.2%, B: 1.5 ± 0.2%, *t*(33) = 3.18, *p* = 0.003], but not for the right hand [both A and B: 1.6 ± 0.2%, *t*(33) = 0.69, *p* = 0.50]. Finally, a three-way interaction was observed for group, hand and sequence, but surprisingly, only for between-sequence inter-movement intervals. This effect is the opposite of the effect on the number of correct sequences. While for the Int group, there is not a significant difference between improvement on between-sequence inter-movement intervals between LH and RH, for either sequence, for the NoInt group, a significant difference is observed only for the B sequence [LH: 5.5 ± 1.3%, RH: 6.1 ± 1.3%, *t*(16) = -2.26, *p* = 0.038] but not for the A sequence.

**Table 2:** Results of the mixed design ANOVA examining the transfer conditions for four components contributing to overall improvement in performance.

Factor	Quantity	*F*(1,31)=	*p*
Sequence (A/B)	Within-sequence IMI	78.7	<0.001*
	Between-sequence IMI	13.24	0.001*
	Movement time	16.24	<0.001*
	Touch times	6.98	0.013*
Hand (L/R)	Within-sequence IMI	9.88	0.004*
	Between-sequence IMI	0.52	0.48
	Movement time	1.65	0.21
	Touch times	0.15	0.7
Group (Int/NoInt)	Within-sequence IMI	0.11	0.74
	Between-sequence IMI	0.73	0.4
	Movement time	0.04	0.85
	Touch times	0.17	0.68
Group × Sequence	Within-sequence IMI	4.66	0.039*
	Between-sequence IMI	1.48	0.23
	Movement time	0.001	0.97
	Touch times	3.05	0.091
Group × Hand	Within-sequence IMI	2.78	0.11
	Between-sequence IMI	0.25	0.62
	Movement time	0.13	0.72
	Touch times	0.02	0.90
Hand × Sequence	Within-sequence IMI	5.17	0.03*
	Between-sequence IMI	0.01	0.93
	Movement time	2.88	0.10
	Touch times	7.17	0.012*
Group × Hand × Sequence	Within-sequence IMI	3.09	0.089
	Between-sequence IMI	7.80	0.009*
	Movement time	1.60	0.22
	Touch times	3.73	0.063

*All values are normalized. Within-subject factors were sequence (A or B) and hand (left or right), with a between subject factor of group (Int or NoInt). IMI signifies inter-movement interval. Significant results (p < 0.05) are marked with an asterisk (^∗^).*

### Transfer – Novelty Effect

Although robust changes occur on a time scale of hours and days, important changes in performance occurred on a much shorter time scale. Whenever participants were introduced to a new task condition, the level of performance depended on: (1) previous experience with the task (including experience with a different hand or a different sequence of movements), thus all transfer conditions were significantly better than the pre-test; (2) the actual experience (familiarity) with any specific task condition, reflected in the changes in performance – in novel, un-familiar conditions there were very rapid block-by-block gains (i.e., how much learning takes place during the four 30 s tests). To quantify the novelty effect, slopes of linear regression lines that were fitted to each of the four test blocks were calculated, with a steeper slope indicating greater novelty, shown in **Figure 3C** (as in Korman et al., 2003). At 24 h re-test, the novel conditions are the conditions in which no specific training was given: the B sequence produced with the right hand in the two experimental groups, and the B sequence produced with the left hand in the NoInt group (**Figure 3C**), but not for the Int group. For the NoInt group, the slopes for the two transfer conditions were greater than for the trained sequence [paired *t*-tests, B: *t*(16) = -5.22, *p* < 0.001; B RH: *t*(16) = -5.06, *p* < 0.001], whereas for the interference group, the slope for the left hand B test was not significantly different to the trained sequence [*t*(16) = -1.45, *p* = 0.17], while the slope for the right hand was significantly different [B RH: *t*(16) = -3.81, *p* = 0.002].

Altogether, the analysis of transfer of performance gains, as well as of novelty effect, indicate differences in the representations of sequence A and sequence B as a function of interference experience at 24 h after initial training.

## Discussion

How does interference impact learning-related changes in performance? We studied the effect of behavioral retroactive interference on learning a given motor sequence in terms of a set of specific temporal and kinematic features, individually contributing to the acquisition, stabilization and offline improvement. All participants practiced a sequence of finger-opposition movements; half subsequently practiced a second competing sequence after an interval of 90 min. Participants were retested 24 h post-training after a night sleep. As was previously shown (Korman et al., 2007), training on the interfering task within 2 h after the initial training session prevented the expression of any delayed gains in terms of the number of correct sequences by 24 h post-training, while the absolute accuracy of performance was not affected by the interference experience. By decomposing the overall improvement in performance into individual causes (Friedman and Korman, 2012), kinematic and temporal (**Figure 4**), we provide new evidence for two types of processes contributing to the offline, delayed, consolidation of the trained sequence: an interference-sensitive optimization of inter-movement intervals and an interference-independent optimization of touch times, movement velocity and movement amplitude. We conjecture that overall learning, as measured by changes in common behavioral indices of number of correct sequences and accuracy of performance, is a sum of dissociable processes, differentially contributing to the optimization of motor behavior over 24 h window post-training, and highly sensitive to post-training interference experiences.

### Differential Contribution of Temporal vs. Kinematic Components to Improvement in Performance

In line with the literature on factors promoting motor learning (Flanagan et al., 2006), we suggest that movement-related changes (larger amplitude/larger velocity) may reflect an enrichment of the amount of sensory feedback available, i.e., bottom-up learning processes. This increase in sensory information may contribute to the accuracy of the timing, as has been observed in tapping studies (Aschersleben and Prinz, 1995; Drewing et al., 2002). In contrast, the timing-related changes (inter-movement intervals) are likely to be a result of higher order representations of the sequence, a “motor plan” (Hikosaka et al., 2002). Thus, behavioral retroactive interference probably disrupts only these more abstract, organization driven components of the overall skill acquisition.

In this study, we selected a more complex FOS task (Karni, 1996), which requires larger finger movements, taking more time and coordination of multiple effectors while in our previous study (Friedman and Korman, 2012), with a keyboard task, the movements accounted for a very small proportion of the overall sequence time. The FOS task allows subjects to select individual performance strategies, as the movements can be produced from the large number of degrees of freedom available. Nevertheless, as in the keyboard version of the task, the within-session improvement in overall number of correct sequences was mainly due to reducing inter-movement intervals rather than decreasing the duration of the individual finger movements. This finding suggests that in novel motor sequence learning, the process of improvement is governed predominantly by strategy-related factors (planning of sequence and timing of movements) rather than biomechanical optimization (improvement in muscle synergies and decreasing the degrees of freedom involved in movement production). According to Rozanov et al. (2010), long-term training will also lead to changes in the biomechanical organization of the movements.

Previous studies, predominantly utilizing serial reaction time keyboard tapping tasks, suggested that sequences of actions tend to organize into chunking patterns (Gobet et al., 2001; Verwey, 2003; Kennerley et al., 2004), reflecting the representation of the trained motor sequence as if it consisted of several short, up to 6–7 element sub-sequences. In the FOS paradigm used in the current study, five movements comprised a sequence, which was produced repetitively during a 30 s test. Within-sequence inter-movement intervals decreased to approximately zero during acquisition and post-training phases of learning, suggesting temporal optimization of the trained sequence as a whole. Between-sequence inter-movement intervals also decreased, despite the fact that the same movement of the little finger constitutes the start and end element of the sequence. We found that the consolidation processes of within-sequence inter-movement intervals are sensitive to interference, whereas the between-sequence inter-movement interval consolidation is not sensitive to interference. This difference suggests that consolidation processes may contribute to the evolution of a five-element sequence as a chunk, if allowed. Within-sequence inter-movement intervals (chunking patterns) also appear to be effector-dependent, in line with previous findings (Miyapuram et al., 2006).

### Interference

Pre and post-training treatments (physical, pharmacological, and behavioral) have been shown to selectively affect (enhance or impair) motor learning and memory consolidation (Nitsche et al., 2003; Fischer et al., 2005; Korman et al., 2007). There is an implication of a control mechanism that needs to be set and scheduled for learning to be consolidated. The interference effects occur after the termination of training in the awake state, suggesting that development of “immunity” to interference is a time-dependent process that takes at least 5 h post-training to evolve (Korman et al., 2003, 2007). Here, we showed that retroactive interference is disruptive only for a subset of mnemonic processes underlying motor skill acquisition. The within-sequence inter-movement intervals were changed and optimized offline only in the NoInt group. Purely motor executive aspects of finger movements (touch times, velocity, and amplitude of individual movements) changed in both groups regardless of the interference experience, i.e., similar patterns were observed in the interference and non-interference groups.

We note that our findings may not be universal. Studies using different types of motor sequence tasks (e.g., making out-and-back movements in a given sequence) have shown that interference effects differ between explicit learning (i.e., learning the order of the sequence) compared to implicit aspects, such as the spatial accuracy or movement time (Ghilardi et al., 2009). These implicit aspects of performance improved even during random sequences (Nissen and Bullemer, 1987; Ghilardi et al., 2009; Wong et al., 2015), suggesting that these aspects of motor learning are independent from sequence-dependent learning. We note that sequential finger opposition tasks differ from out-and-back movements in several important ways. As there is generally a fixed, externally cued duration between subsequent movement onset times in the out-and-back tasks (typically 1 s), slower movements are beneficial (as they can improve accuracy), whereas improvement in the finger opposition task requires reducing times between movement onset of subsequent movements. Second, the out-and-back task is serial in nature – there is no way to combine movements, whereas overlapping movements (i.e., coarticulation) are beneficial in the finger opposition task. Third, while improving reaction time to the audio cue can improve performance in the out-and-back task, the finger opposition movements are self-initiated. These substantial differences mean that we expect learning to manifest itself differently in the two tasks.

### Transfer

Our results suggest that interference training on a competing sequence B not only caused underdeveloped learning of the initially trained sequence A, but improved the performance of sequence B, as was seen in the various interactions observed (also see **Figure 3B** – in generalization of performance gains, **Figure 3C** – in novelty effect). This was presumably achieved by rerouting the plasticity resources to establish the long-term representation of the competing sequence [in line with behavioral tagging theory (Moncada et al., 2015)]. Thus, the representation of the trained task after interference training was qualitatively, not only quantitatively different as compared to the no-interference condition. Non-specific transfer of task-general parameters was also observed, all transfer tests were significantly different from baseline performance of the untrained sequence (as previously observed in Korman et al., 2003), reflecting improvements in task components that are not dependent on knowledge of the sequential structure, such as improvements in stimulus–response mapping, or motor command generation (Müssgens and Ullén, 2015). Indeed, changes in kinematic parameters (e.g., amplitude and peak velocity) showed similar changes between the groups, regardless of the interference experience. Altogether, our findings are in line with recent notions of dissociable hierarchical processes, with each level exhibiting its own time course and susceptibility to generalization across end effectors and task (Kornysheva and Diedrichsen, 2014; Diedrichsen and Kornysheva, 2015). Following a single training it is plausible to assume that sequence-specificity of task representation is not entirely formed, and exists at an intermediate level (between selection and execution), allowing partial transfer to the other hand. Multiple trainings are required to achieve robust sequence-specificity (Korman et al., 2003).

### Limitations

There are several limitations to this study that should be noted. First, the participants performed the tasks with magnetic motion capture sensors taped to their fingernails. While these sensors are light (3.7 g), this weight is not insignificant compared to the average weight of the distal phalange (approximately 3 g) (Friedman and Flash, 2009). Further, although the sensors were taped so as to leave the fingerpads unencumbered, the tape on the fingers, and palm, and the pull of the cables would likely have affected the kinematics of the movements, which may limit the generalizability of these findings. However, we note that the number of sequences performed is comparable to those found in previous studies (e.g., Korman et al., 2003).

Further, we note that as the first and last movements in the sequence were essentially the same movement (with the little finger), coarticulation as defined here is not possible between sequences. Nevertheless, we obtained robust learning in serial production of the same movement between sequences. However, we cannot determine whether the larger inter-movement intervals between the movements of the little finger are due to longer reaction times observed at the start of a sequence (Kennerley et al., 2004), or due to physical constraints.

## Conclusion

As different types of interference in the form of competing experiences are naturally present in almost any ecological learning setting, their impact on the time course of learning in typical and clinical populations should be considered in the construction of training protocols. Current results suggest that retroactive interference is disruptive only for a subset of mnemonic processes underlying motor skill consolidation. Thus, the magnitude of the devastating effect of competing experience on delayed offline gains, such as the number of correct sequences, may be crucially dependent on the particular underlying components of performance that are emphasized in task demands. While optimization of the timing of motor components is likely to be interference sensitive, biomechanical or sensory-feedback related optimization is likely to be interference independent. This implies that in clinical cases, for example cases with constraints on biomechanics such as muscle weakness or limited range of motion, improvement in terms of temporal organization can still be expected if encouraged by appropriate protocol design, e.g., feedback on compliance with the temporal structure of the sequence.

## Author Contributions

JF and MK conceived and designed the experiments. JF analyzed the raw data. JF and MK participated in the statistical analysis and interpretation of the data. JF and MK wrote the article.

## Conflict of Interest Statement

The authors declare that the research was conducted in the absence of any commercial or financial relationships that could be construed as a potential conflict of interest.
